# Application of 18F-FDG PET-CT Images Based Radiomics in Identifying Vertebral Multiple Myeloma and Bone Metastases

**DOI:** 10.3389/fmed.2022.874847

**Published:** 2022-04-18

**Authors:** Zhicheng Jin, Yongqing Wang, Yizhen Wang, Yangting Mao, Fang Zhang, Jing Yu

**Affiliations:** ^1^Department of Nuclear Medicine, Second Affiliated Hospital, Dalian Medical University, Dalian, China; ^2^School of Geophysics and Information Technology, China University of Geosciences, Beijing, China

**Keywords:** radiomics, multiple myeloma, bone metastases, 18F-FDG PET-CT, SUVmax

## Abstract

**Purpose:**

The purpose of this study was to explore the application of 18F-fluorodeoxyglucose positron emission tomography/computed tomography (18F-FDG PET/CT) image radiomics in the identification of spine multiple myeloma (MM) and bone metastasis (BM), and whether this method could improve the classification diagnosis performance compared with traditional methods.

**Methods:**

This retrospective study collected a total of 184 lesions from 131 patients between January 2017 and January 2021. All images were visually evaluated independently by two physicians with 20 years of experience through the double-blind method, while the maximum standardized uptake value (SUVmax) of each lesion was recorded. A total of 279 radiomics features were extracted from the region of interest (ROI) of CT and PET images of each lesion separately by manual method. After the reliability test, the least absolute shrinkage and selection operator (LASSO) regression and 10-fold cross-validation were used to perform dimensionality reduction and screening of features. Two classification models of CT and PET were derived from CT images and PET images, respectively and constructed using the multivariate logistic regression algorithm. In addition, the ComModel was constructed by combining the PET model and the conventional parameter SUVmax. The performance of the three classification diagnostic models, as well as the human experts and SUVmax, were evaluated and compared, respectively.

**Results:**

A total of 8 and 10 features were selected from CT and PET images for the construction of radiomics models, respectively. Satisfactory performance of the three radiomics models was achieved in both the training and the validation groups (Training: AUC: CT: 0.909, PET: 0.949, ComModel: 0.973; Validation: AUC: CT: 0.897, PET: 0.929, ComModel: 0.948). Moreover, the PET model and ComModel showed significant improvement in diagnostic performance between the two groups compared to the human expert (Training: *P* = 0.01 and *P* = 0.001; Validation: *P* = 0.018 and *P* = 0.033), and no statistical difference was observed between the CT model and human experts (*P* = 0.187 and *P* = 0.229, respectively).

**Conclusion:**

The radiomics model constructed based on 18F-FDG PET/CT images achieved satisfactory diagnostic performance for the classification of MM and bone metastases. In addition, the radiomics model showed significant improvement in diagnostic performance compared to human experts and PET conventional parameter SUVmax.

## 1. Introduction

Multiple myeloma (MM) was a malignant clonal cell tumor that originated from bone marrow plasma cells. MM extensively invades bone marrow, bones, and extramedullary organs, leading to prime syndromes such as bone pain, anemia, infection, fractures, and kidney damage (1). Bone metastasis(BM) was a common event in tumor progression. The common primary tumors were lung cancer, breast cancer, and prostate cancer (2). The spine contained a rich blood supply and was also the most frequent site to be involved. MM and BM had different pathogenesis, but the site of occurrence, clinical manifestations, and imaging features were similar, which makes it difficult to distinguish. Lesions that were difficult to characterize were often misdiagnosed as other orthopedic diseases, especially for MM and bone metastases with unknown primary lesions. Misclassifications will significantly affect the quality of patient survival and survival rates due to the variability of treatment options (3, 4). Therefore, it was particularly important to improve the diagnostic accuracy of MM and BM.

Previous studies had considered serologic markers such as serum creatinine, serum globulin, and serum alkaline phosphatase as crucial information for differentiating MM from BM. However, some patients with light chain secretory, low, and non-secretory myeloma may have low or normal levels of these serologic markers, and such examinations were often used for preliminary screening (5, 6). 18F-fluorodeoxyglucose positron emission tomography/computed tomography (18F-FDG PET/CT) images combined anatomical and metabolic information to provide relatively high sensitivity and specificity to assess bone damage and detect extramedullary lesions (7, 8). In patients with early MM, up to 40% of patients could detect additional lesions by PET/CT examination to guide individualized treatment plans (9). The International Myeloma Working Group had reached a consensus and recommended 18F-FDG PET/CT as one of the best imaging methods for the examination of MM and other plasma cell diseases (10). However, there still exist lesions that were difficult to identify even for experienced physicians in clinical work, especially osteolytic lesions (11, 12).

Radiomics converted texture, intensity, density, and other features extracted from medical images into mineable high-dimensional data through automated or semi-automated methods, which could be used as a non-invasive assessment of spatial heterogeneity of tumors and facilitate personalized patient treatment (13, 14). The performance of radiomics analysis had been demonstrated in previous studies to identify cancer types, predict treatment efficacy, and predict disease progression (15–17). In addition, radiomics had shown unique advantages in molecular areas such as prediction of cancer gene expression and lymph node metastasis (18, 19). However, previous radiomics mostly focused on CT and MRI, and the diagnostic value of radiomics combined with 18F-FDG PET/CT for MM and BM was still unclear (20, 21).

The purpose of this study was to explore the feasibility of radiomics based on 18F-FDG PET/CT images in the identification of MM and BM and whether it could improve the diagnostic performance of these two diseases.

## 2. Materials and Methods

### 2.1. Patients

**Figure 1:**
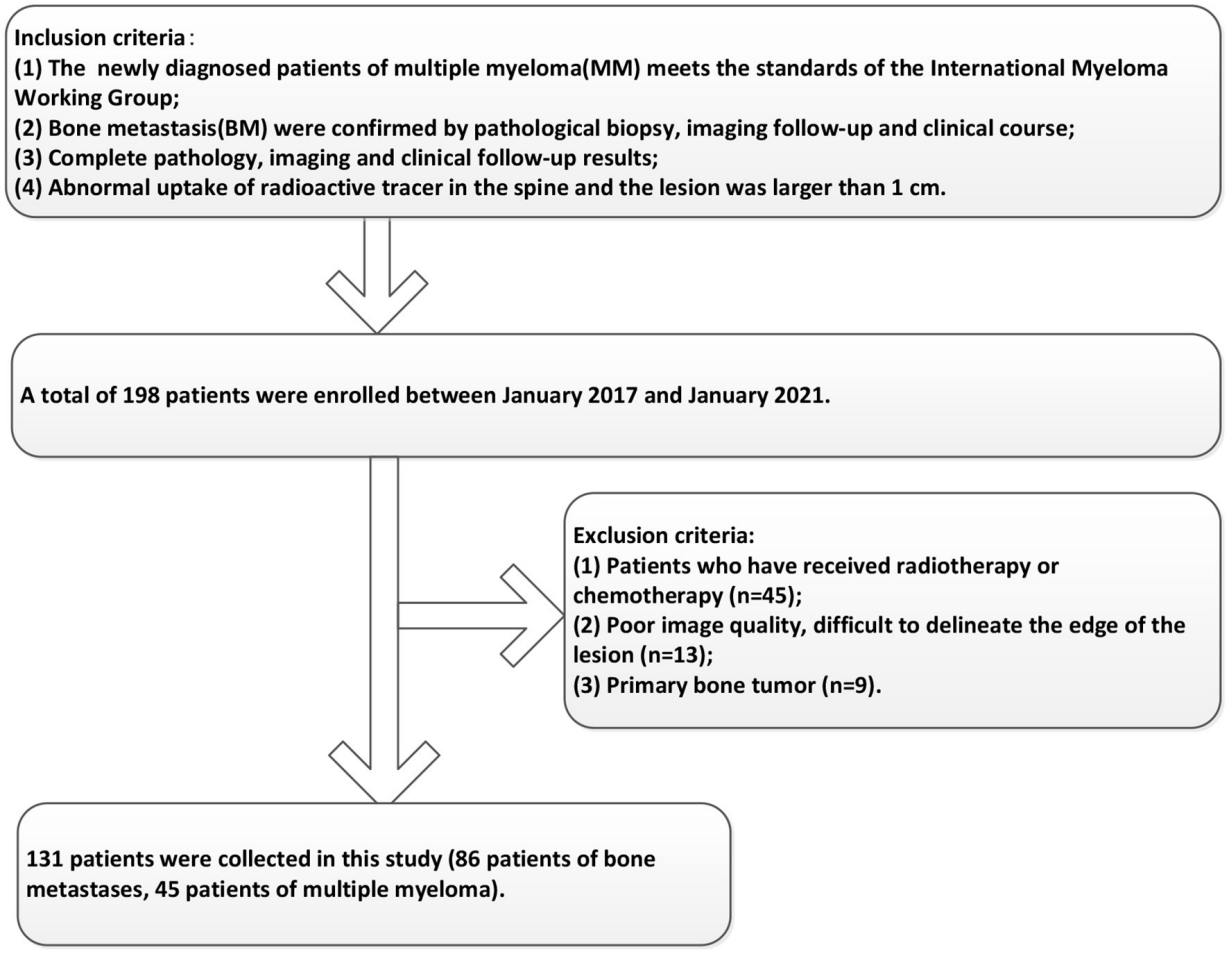
The enrollment criteria of the patients in this study.

Participants between January 2017 and January 2021 were enrolled in this study according to the following inclusion criteria: (1) The diagnosis of MM meets the standards of the International Myeloma Working Group (22); (2) BM were confirmed by pathological biopsy, imaging follow-up, and clinical course; (3) Complete pathology, imaging, and clinical follow-up results; (4) Abnormal uptake of radioactive tracer in the spine and the lesion was larger than 1 cm. In addition, the exclusion criteria included the following: (1) Patients who have received radiotherapy or chemotherapy; (2) Poor image quality, difficulty to delineate the edge of the lesion; (3) Primary bone tumor. The enrolled patients were randomly divided into training groups and validation groups according to the ratio of 7:3 for subsequent model construction. This retrospective study was approved by the hospital's ethical review, and the patient's informed consent requirement was waived. The enrollment criteria of the patients in this study were shown in Figure 1.

### 2.2. Image Protocol

All the image acquisition procedures in this study were completed in the PET/CT(Philips Ingenuity TF). The radioactive tracer 18F-FDG was automatically synthesized by the cyclotron (Sumitomo, Japan) and the 18F-FDG chemical synthesis module (Sumitomo, Japan), and the radioactive tracer purity was guaranteed to be >95%. The patient fasted for at least 6 h before the examination, and the glucose level lower than 11.1 mmol/L was ensured by routine measurement of the blood samples before the PET/CT examination. Patients were injected with 18F-FDG(5.55 MBq/kg) intravenously in a quiet state and were placed in a room with dim light for 40–60 min, and then underwent PET/CT after emptying the bladder. The scanning process includes a low-dose CT scan and PET scan from the top of the skull to the upper thigh. CT acquisition parameters were as follows: tube current tube voltage was automatically generated according to the positioning image, tube rotation time: 0.35 s, output voltage: 70–140 KV, output current: 20–450 mA, layer thickness: 0.7 mm, reconstruction time: 40 frames/s, reconstruction matrix: 512 × 512, number of detector rows: 64, pitch: 0.15–1.5. After standardizing all parameters of the patient's PET/CT images, the window width and window level of the CT images were set to 350 and 50, respectively, and the PET data were reconstructed by attenuation correction and iterative method (Ordered Subsets Expectation Maximization, OSEM), and then transmitted to the MedEx workstation together with the CT images for fusion imaging. The maximum standardized uptake value (SUVmax) was automatically generated by the workstation based on the information of the subject's weight, injection dose, and time. The region of interest (ROI) was outlined along with the extent of the lesion at the level where the concentration of the radioactive tracer was most obvious, and the workstation automatically calculates the SUVmax.

### 2.3. Confirmation of Lesions and Huamn Expert's Qualitative Classification

Considering that detailed pathological examination was not available in all patients, we determined the diagnosis of the lesions on the basis of pathological biopsy, imaging follow-up, and clinical course of the disease. Independent visual analysis of lesions was evaluated by two physicians (TAJ and JY) with 20 years of diagnostic experience using the double-blind method, physicians were not informed of the patient's clinical information and pathology but were told that the lesion was either MM or BM. The weighted kappa analysis was used to determine the interobserver agreement. Kappa coefficients of 0–0.20, 0.21–0.40, 0.41–0.60, 0.61–0.80, and 0.81–1 were considered to be slight, fair, moderate, good, and almost perfect agreement, respectively (23).

### 2.4. Segmentation and Feature Extraction

**Figure 2:**
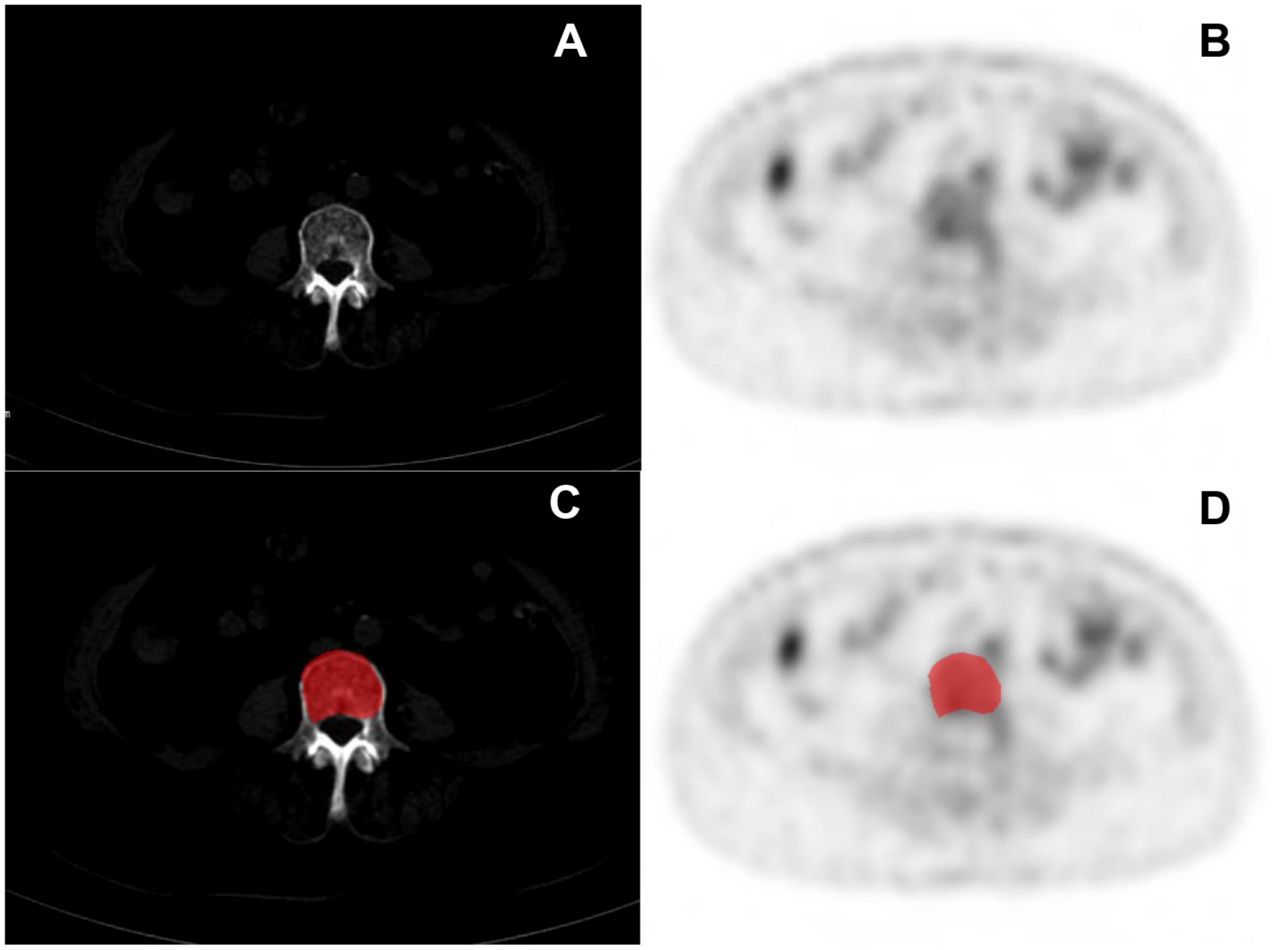
Clinical cases PET/CT images of multiple myeloma (MM) **(A,B)** and the delineation of the region of interest (ROI) **(C,D)**.

Segmentation of the lesions was also performed by double-blind methods with physicians who had 10 years of experience (CMY and ZJN) in diagnostic work. All features were extracted in MaZda software, which has been proven in previous studies for radiomics, and all radiomics features extracted were in accordance with Image Biomarker Standardization Initiative (IBSI) standards (24, 25). The source images were extracted from the hospital PACS workstation and saved in BMP format. The object of this study was the largest cross-sectional area of the vertebral lesion, and the features were extracted by the 2D mode of Mazda software. Before the image was applied to MaZda software for feature extraction, uniform and standardized pre-processing of the image was performed by the method of μ ± 3σ to make the features more reproducible and verifiable. The abnormal uptake of radioactive tracer on the image was used as the initial ROI, and the physician carefully identified the edges of the lesion and progressively outlined the ROI on the PET and CT images along the edge of the lesion. Because of the long examination time, the physician could make minor adjustments to determine the lesion of interest outlined and eliminate the effects of patient movement or expiratory motion. An example of the ROI outline was illustrated in Figure 2. A total of 279 features were extracted for each ROI, which were included in the following six common categories: gray-level histogram (HSLM), gray-level absolute gradient (GRM), gray-level run-length matrix (GLRLM), gray-level co-occurrence matrix (GLCM), autoregressive model (ARM), and wavelet. The interpretation of the features is described in detail in the previous study (26).

### 2.5. Reliability Analysis

To ensure the stability and reproducibility of the extracted features, a reliability test was performed. Another physician with 10 years of diagnostic experience repeated the outlining of the above ROI by randomly selecting 30 lesions. The reliability of the ROI outlined by the two physicians was assessed by the class correlation coefficient. Class correlation coefficients greater than 0.75 for radiomic features were considered to have good stability and reproducibility and were used for subsequent feature screening and model construction.

### 2.6. Dimensionality Reduction and Model Establishment

Before the feature screening, the normalization of the features was performed by the Z-score method, which aims to avoid the training of the model with too small weights, causing numerical instability, and to improve the comparability of the data, while enabling the parameter optimization to converge at a faster rate. After the reliability test, the training group was subjected to the least absolute shrinkage and selection operator (LASSO) regression for further data selection. LASSO regression was performed by fitting a generalized linear model with variable selection and complexity adjustment regularization. The filtering features were validated by 10-fold cross-validation based on the bias minimization criterion. Finally, for the final selected non-zero features, a classification model was built by multivariate logistic regression. CT models and PET models were constructed based on the final selected features (features were derived from CT and PET images, respectively). In order to better evaluate the performance of radiomics, a combined model (ComModel) was constructed by adding the PET conventional parameter SUVmax combined with PET radiomics features.

### 2.7. Model Comparison

The performance of all classification diagnostic models was evaluated by comparing the area under the receiver operating characteristic (ROC) curve (AUC), accuracy, sensitivity, specificity, negative predictive value (NPV), and positive predictive value (PPV), while 95% CI of AUC were calculated. The DeLong test was used to compare the diagnostic effects between the models, and *P* < 0.05 was considered to be statistically different. In addition, calibration curves and Brier scores were used to evaluate the predictive ability and goodness of fit of the classification models to observe the agreement between the actual and predicted probabilities of the models. Decision curve analysis (DCA) was used to visualize and evaluate the clinical net benefit and clinical utility of the classification prediction model by the graphical presentation.

### 2.8. Statistical Analysis

Independent samples *t*-test or Mann—Whitney *U*-test was used to compare continuous variables with normal or non-normal distribution in the MM and BM groups. Categorical variables between the two groups were assessed using the chi-square test or Fisher test and weighted Kappa statistics were used to assess interobserver agreement. The processing of features screen, model construction, and comparison of the diagnostic performance of the models were performed in R software (version 4.1.1) and Python (version 3.8.1). IBM SPSS (version 21.0) and MedCalc software (version 20.0) were used for other clinical data analysis and ROC curve plotting. Probability values of *P* < 0.05 were considered statistically significant.

## 3. Results

### 3.1. Basic Patient Information

According to the inclusion and exclusion criteria, a total of 131 patients were enrolled, including 86 patients who were diagnosed with bone metastases (BM), and the remaining 45 patients were confirmed as MM. The statistics and comparison of basic information of the patients were shown in Table 1. According to the diagnostic criteria of the International Myeloma Working Group, the stages of MM(ISS standard) were as follows: stage I: *n* = 10; stage II: *n* = 19; stage III: *n* = 16. The primary tumor of patients diagnosed with BM were as follows: lung cancer: *n* = 24; breast cancer: *n* = 17; prostate cancer: *n* = 14; pancreatic cancer: *n* = 4; kidney cancer: *n* = 3; ureteral cancer: *n* = 3; stomach cancer: *n* = 3; thyroid cancer: *n* = 3; liver cancer: *n* = 2; bladder cancer: *n* = 2; colon cancer: *n* = 2; parotid cancer: *n* = 2; gallbladder cancer: *n* = 2: fallopian tube cancer: *n* = 2; uroepithelial cancer: *n* = 1; esophagus cancer: *n* = 1; cervical cancer: *n* = 1. A total of 184 lesions were obtained and randomly divided into training and validation groups according to the ratio of 7:3 (The training group: BM: *n* = 80, MM: *n* = 49: The validation group: BM: *n* = 34, MM: *n* = 21).

**Table 1:** Basic information for patients in the training and validation cohorts.

	**The training cohort**			**The validation cohort**		
	**BM**	**MM**	***P***	**BM**	**MM**	***P***
**Gender**			0.171			0.079
Female	22	16		11	8	
Male	38	15		15	6	
**Age**	63.58 ± 12.07	58.71 ± 10.08	0.470	60.88 ± 11.15	57.79 ± 13.20	0.521
Range	33–90	43–75		37–86	34–77	
**Lesion form**			0.057			0.101
Osteolytic	52	37		22	18	
Osteoblastic	15	2		6	0	
Mixed	13	10		6	3	
**ISS stage**						
I	-	10		-	3	
II	-	23		-	13	
III	-	16		-	5	
**Extramedullary mass**	27	19	0.604	11	7	0.778
**SUVmax**	6.84 ± 3.32	4.06 ± 1.61	<0.001	6.79 ± 3.31	4.38 ± 1.60	0.001
**Osteoporosis**			0.001			0.007
Postive	17	33		8	13	
Negative	63	16		24	8	
**Confirmation**			0.001			0.001
Biopsy	29	49		14	21	
Follow-up	51	0		18	0	

*P < 0.05 was considered to be statistically significant; BM, bone metastases; MM, multiple myeloma; Extramedullary mass, Extramedullary soft tissue mass; ISS stage, International Staging System classification*.

### 3.2. Feature Selection, Model Establishment, and Validation

**Figure 3:**
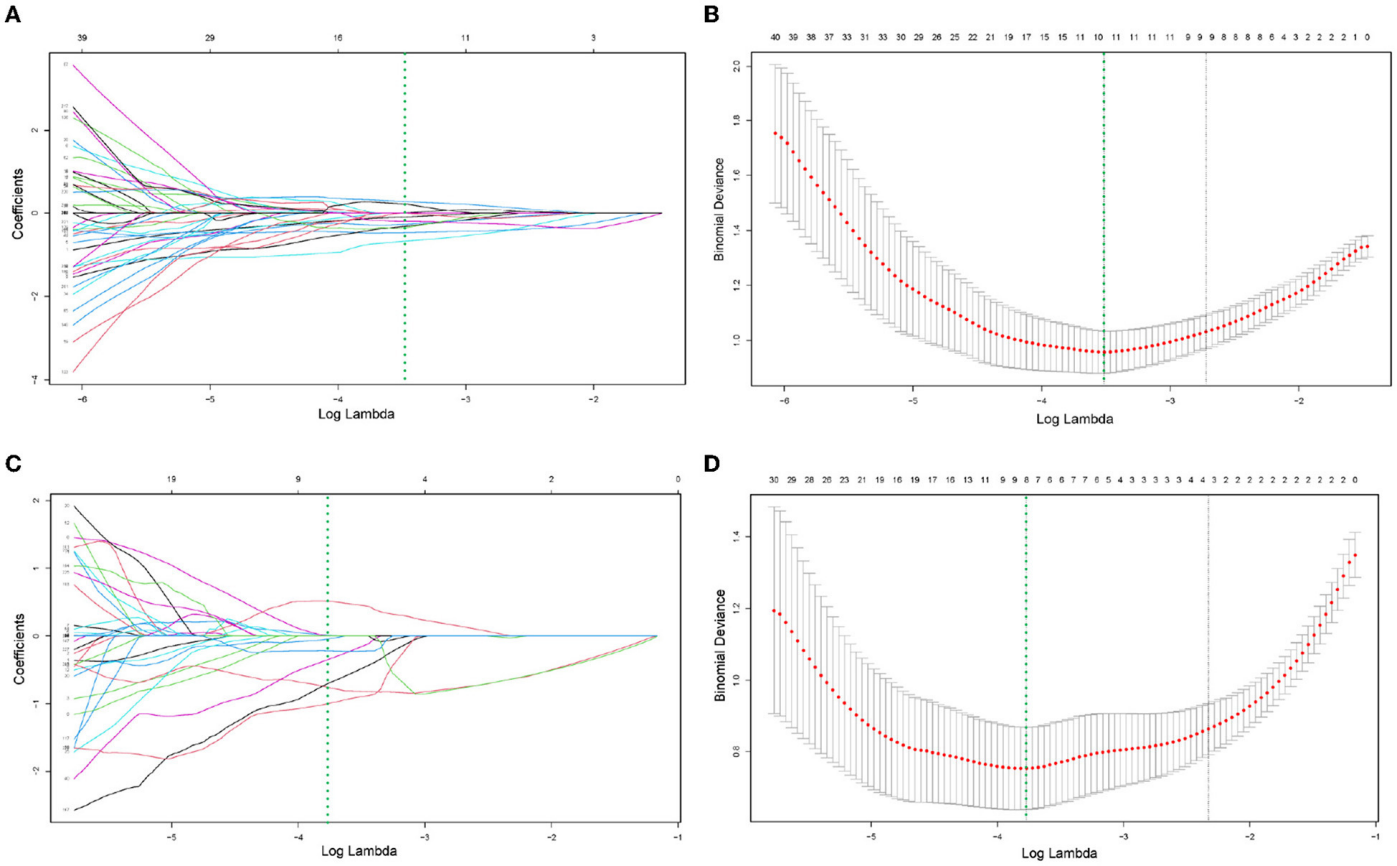
**(A–D)** Demonstrated the specific process of least absolute shrinkage and selection operator (LASSO) regression analysis screening features for CT model and PET model, respectively. **(A,C)** Showed process of features selection. The vertical green line was plotted at the optimal λ of 0.030 and 0.023 for CT and PET model, respectively. Ten and eight features with non-zero coefficients were finally selected for CT and PET model, respectively. **(B,D)** Showed that features selection performed by 10-fold cross-validation with the criterion of minimum deviance.

After reliability testing and excluding features with ICC coefficients less than 0.75, 223 and 234 radiomics features were extracted from CT and PET in the training group, respectively. Then, 10 and 8 texture features were obtained from CT and PET after LASSO regression and 10-fold cross-validation, respectively. The LASSO regression screening process was described in detail in Figure 3, and the final filtered feature information in the training and validation groups of the MM group and BM group was illustrated in the heat map in Supplementary Figures S1–S4, which the differences in feature expression between the MM and BM groups were clearly seen. Furthermore, the selected features of CT and PET coefficients were also described in Supplementary Table A. In the training group, all models achieved very high AUC values and the classification diagnostic performance of the ComModel (AUC:0.973; CI95%:0.928–0.993) was significantly improved compared to the CT (AUC:0.909; CI95%:0.846–0.952) and PET models (AUC:0.949; CI95%:0.896–0.980) (*P* = 0.013 and *P* = 0.024, respectively), while the PET model did not show a statistical difference in the DeLong test although it had a higher diagnostic performance compared to the CT model (*P* = 0.131). In the validation group, the ComModel (AUC: 0.948; CI95%: 0.853-0.990) and the PET model (AUC:0.929; CI95%: 0.826–0.981) achieved similar diagnostic performance and outperformed the CT model (AUC:0.897; CI95%: 0.785–0.963), and the DeLong test suggested no statistical difference between the three models (*P* = 0.309, *P* = 0.466, and *P* = 0.496, respectively).

### 3.3. Diagnostic Performance Between the CT Model, PET Model, ComModel, Human Experts, and SUVmax

**Figure 4:**
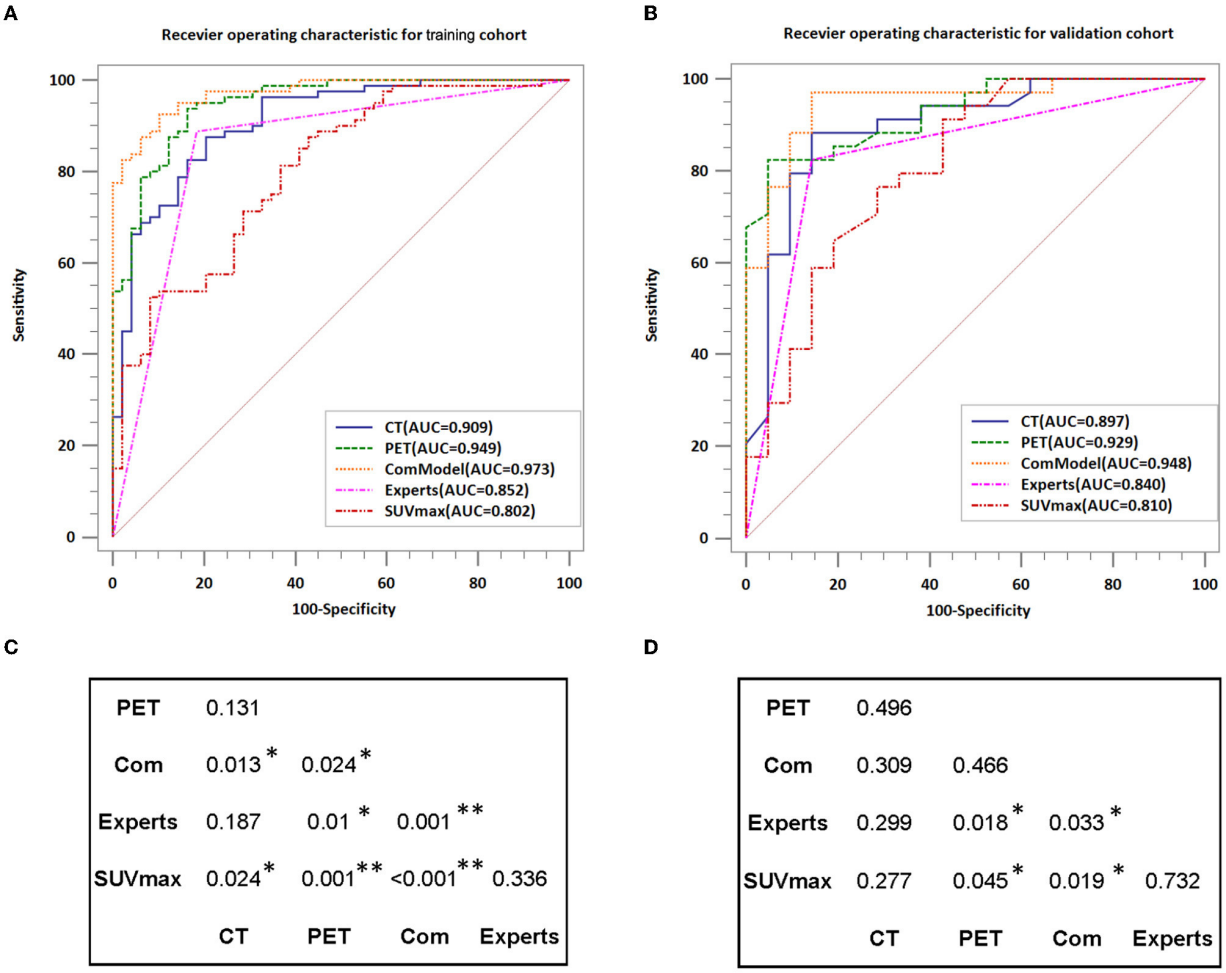
Comparison of the diagnostic performance of different models; **(A,B)** Show the ROC curves for each model in detail while also recording the value of AUC. **(C,D)** Indicate the results of the DeLong test for the training group and validation group, respectively. AUC, area under the curve; *P < 0.05; **P < 0.01.

Human experts' classification diagnostic of lesions was estimated by the kappa coefficient, and in this study, the weighted *k*-value for the interobserver agreement was 0.832, which indicates a relatively reliable agreement. In the training and validation groups, the AUC values of human experts for the classification and diagnosis performance of MM and BM were 0.835 (CI95%:0.760–0.895) and 0.840 (CI95%:0.717–0.925), respectively. while the AUC values of SUVmax between the two groups were 0.802 (CI95%:0.723–0.867) and 0.810 (CI95%:0.681–0.903), respectively. Both the ComModel and the PET model showed significant differences in the classification diagnosis of MM and BM compared to human experts in both the training (*P* = 0.001 and *P* = 0.01, respectively) and validation groups (*P* = 0.033 and *P* = 0.018, respectively). The CT model was not statistically different between the two groups compared to the human experts (*P* = 0.187 and *P* = 0.299, respectively). The ComModel and the PET model also showed great superiority compared to SUVmax between the two groups (Training group: *P* < 0.001 and *P* = 0.001; Validation group: *P* = 0.019, *P* = 0.045). No statistical difference was observed that the human expert compared to SUVmax between the two groups (*P* = 0.036 and *P* = 0.732). The classification diagnostic performance of all models was described and illustrated in Table 2, and the ROC curves of all classification models were illustrated in Figure 4, in addition, the detailed results of the DeLong test were also recorded in Figure 4.

**Table 2:** The diagnostic ability of each model for discriminating vertebral multiple myeloma (MM) from bone metastasis (BM).

	**AUC**	**Accuracy**	**Sensitivity**	**Specificity**	**PPV**	**NPV**
**CT model**						
training cohort	0.909(0.846–0.952)	0.829	0.875	0.796	0.889	0.735
validation cohort	0.897(0.785–0.963)	0.836	0.882	0.857	0.882	0.762
**PET model**						
training cohort	0.949(0.896–0.980)	0.884	0.937	0.837	0.900	0.857
validation cohort	0.929(0.826–0.981)	0.873	0.824	0.952	0.882	0.857
**ComModel**						
training cohort	0.973(0.928–0.993)	0.915	0.925	0.898	0.925	0.898
validation cohort	0.948(0.853–0.990)	0.891	0.971	0.857	0.912	0.857
**Human experts**						
training cohort	0.835(0.760–0.895)	0.845	0.875	0.796	0.875	0.796
validation cohort	0.840(0.717–0.925)	0.836	0.824	0.857	0.824	0.857
**SUVmax**						
training cohort	0.802(0.723–0.867)	0.729	0.875	0.571	0.775	0.653
validation cohort	0.810(0.681–0.903)	0.745	0.912	0.571	0.794	0.667

*AUC, the area under the ROC curve; PPV, postive predictive value; NPV, negative predictive value*.

### 3.4. Clinical Use and Calibration

**Figure 5:**
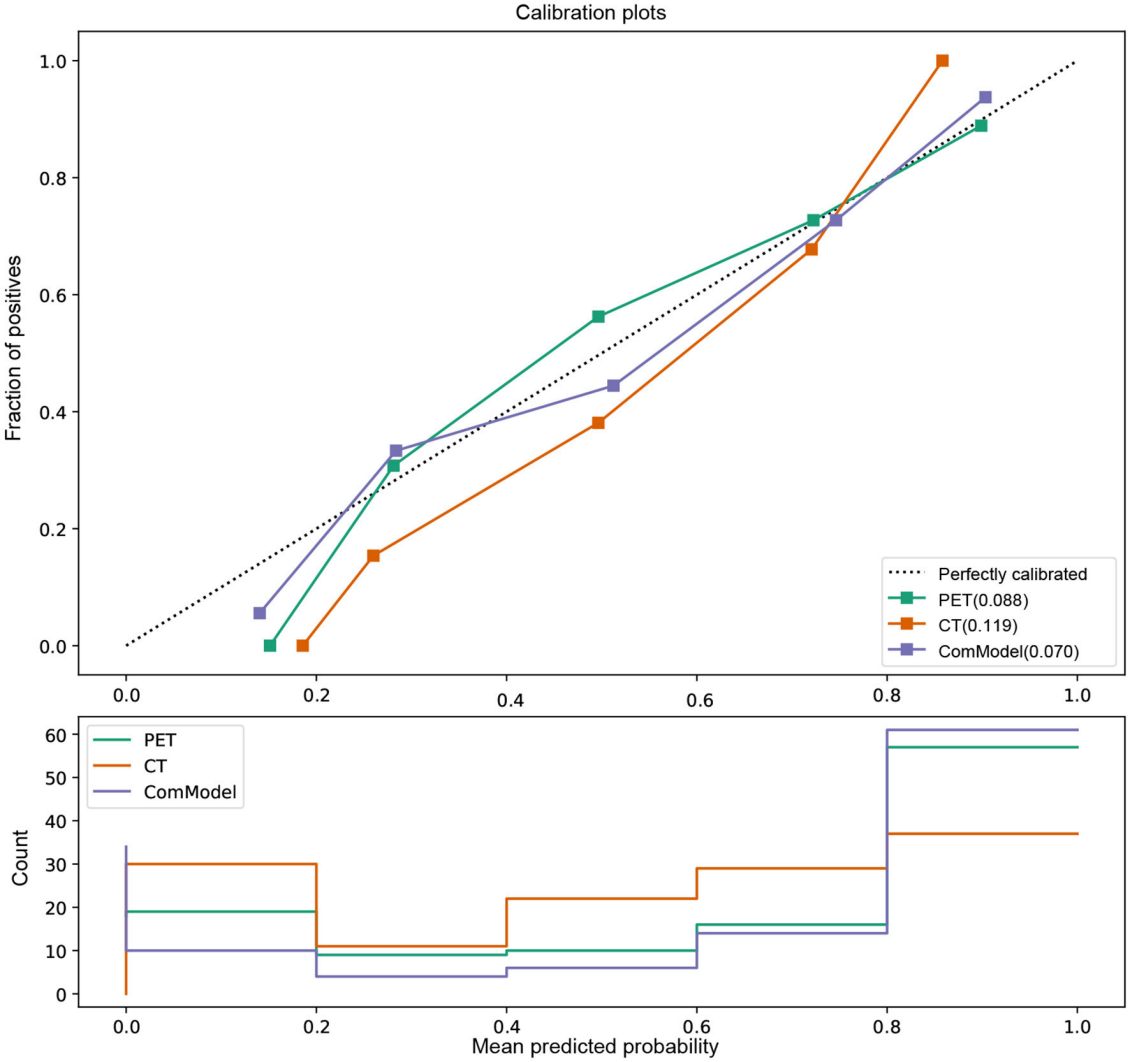
Comparison of the calibration curve and Brier score of different models. All three model's calibration curves were closed to ideal curves, indicating that the models had good fitness and predictive ability. The ComModel had better goodness of fit compared to the PET and CT models because of the smaller Brier scores (Brier scores were 0.070, 0.088, and 0.119, respectively). The following figure shows the distribution of the probability of diagnosis for different models.

**Figure 6:**
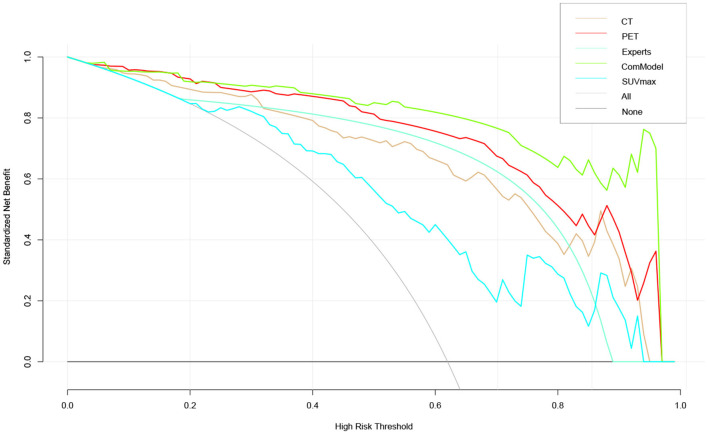
The clinical practicability of the models in this study was evaluated and compared, which indicated that the PET model and the ComModel had better net clinical benefit than the other models.

According to the calibration curves, all the radiomics models were closer to the ideal curve, implying a good categorical diagnostic performance. In addition, the ComModel had a better fitness compared to the PET and CT models because of the smaller Brier scores (Brier scores were 0.070, 0.088, and 0.119, respectively), the calibration curve was shown in Figure 5. In terms of the net clinical gain of the models, both the ComModel and the PET model achieved good net clinical gain and outperformed the other models, the decision curve was shown in Figure 6.

## 4. Discussion

Both myeloma and metastases were common malignant lesions of the spine. When patients only present with lumbar pain and no previous history of tumor, the clinical symptoms and imaging manifestations of both were similar. However, the treatment and prognosis of them were significantly different. Although bone biopsy was the gold standard for identifying benign and malignant lesions, there were limitations in clinical diagnosis due to its invasive examination. Radiomics provided a non-invasive assessment of the lesion and its microenvironment and allow quantification of the spatial heterogeneity of the lesion, which allows identification and evaluates the prognosis. In this study, we constructed and validated a radiomics model based on 18F-FDG PET-CT images and achieved excellent performance in classifying and diagnosing BM and MM. Furthermore, the radiomics model showed unique superiority and clinical utility compared to human experts as well as PET conventional parameter SUVmax. This will play a decisive role as a non-invasive and easy-to-use method in the diagnosis, staging, and re-staging of diseases and even in the selection of treatment strategies for diseases.

Previous studies had pointed out that the traditional imaging features of bone destruction in myeloma involved a series of small focal-like, worm-like, and broad bone destruction, especially for the imaging features such as chisel-like changes in the skull and broad bone destruction in the ribs were specific (27, 28). In addition, most patients with MM showed different degrees of osteoporosis and rarely osteoblastic bone changes. The imaging of bone metastases was characterized by the tendency to invade the pedicle rather than the vertebral body and lack of involvement of extremity bones (29). Mutlu et al. suggest that features such as more sclerotic margins around BM lesions and sharper margins around MM lesions may also be used to differentiate among them (12). Although these studies indicate that these features may be crucial information to identify them, there were still exist similar imaging presentations of bone metastases or atypical lesions in clinical work. Moreover, MM and BM cannot be discriminated simply from bone destruction. In this study, physicians successfully identified all osteoblastic lesions in both the training and validation cohort of the BM group. However, 17.1% (22/129) of the lesions were incorrectly identified in the classification of osteolytic lesions. Physician identification of lesions on conventional imaging mainly was attributed to subjective visual assessment as well as diagnostic experience. Still, this approach was undoubtedly challenging for younger physicians with less diagnostic experience. Accurate classification of BM and MM was crucial as it relates to the plan of individualized treatment, reduction of complications, and improvement of prognosis.

Maximum standardized uptake value as the conventional parameter of PET/CT was used in past studies to determine the treatment sensitivity and prognostic value of malignant lymphoma in the early and intermediate stages (30, 31). On the other hand, the study of Polat et al. confirmed the predictive value of SUVmax for grading and staging of renal clear cell carcinoma and the risk of stratification (32, 33). In our study, SUVmax in the MM group (SUVmax: 4.06 ± 1.61) compared to the BM (SUVmax: 6.84 ± 3.22) group showed a significant decrease in both the training and validation groups, and similar results had been reported several times in the past studies (34, 35). However, the SUVmax for MM and BM in study Li et al. was (1.6 ± 0.7 and 5.5 ± 2.7), respectively, and this variability may be due to the subjects of that study being derived from 334 patients with 8,896 lesions throughout the body, whereas our study focused on the spine and maximum of two lesions per case (5). There are no clear diagnostic thresholds for SUVmax to identify MM and BM. Furthermore, the AUC values of SUVmax for discriminating the BM and MM in the training and validation groups in our study were 0.802 and 0.810, and the accuracy were 0.729 and 0.745, respectively, which achieved only moderate diagnostic efficacy and were not sufficient to make accurate predictions for the classification of lesions. Furthermore, it was difficult for SUVmax to provide a comprehensive description of the heterogeneity and spatial consistency of lesions.

Radiomics could transcend subjective visual assessment to provide an objective evaluation of lesion and tissue heterogeneity, which served as a new tool to provide valuable information about the microenvironment of lesions that cannot be observed by the human eyes. PET/CT radiomics was demonstrated several times in past studies to play an essential role in the diagnosis and prognosis of diseases and performing assessment of therapeutic efficacy. In our research, the radiomics models constructed based on PET/CT images had high diagnostic efficacy in discriminating MM and BM not only in the training group, with AUC values of 0.909, 0.949, and 0.973 for the CT model, PET model, and ComModel, respectively, but also in the validation group, with AUC values of 0.897, 0.929, and 0.948 for the CT model, PET model, and ComModel, respectively. In addition, the diagnostic performance and clinical utility of the radiomics model were superior to those of the human expert and SUVmax, with incremental value for differential and diagnostic purposes, especially the PET and COM models. It should note that a proportion of patients incorrectly staged by human experts (10.9%) and SUVmax (23.9%) were correctly diagnosed by our radiomics model, indicating that the radiomics model could complement the current staging scheme. More importantly, our findings suggest that the PET model had a higher value than conventional CT radiomics in discriminating MM from BM. Although there was no statistical difference in the Delong test, the AUC, Accuracy, Sensitivity, and Specificity of the PET model were significantly improved compared with the CT model. This may be due to the fact that PET images represent radioactive tracer uptake and metabolic information of the lesion. At the same time, PET/CT radiomics reflected the quantification of tumor uptake heterogeneity and earlier detection of lesions compared to conventional imaging, which brings additional value for lesion and tissue specificity identification.

Our results show that Perc.01% and Vertl_RLNonUni were the most representative features in identifying MM and BM, as they appeared in both CT and PET models. The radiomics parameter Perc.01% derived from HSLM reflected the brightness value of the area where it was located and the number of pixels, which further confirmed the excellent performance of the radiomic model since these parameters were closely related to bone density in CT images and radioactive tracer uptake in PET images, as well as to the osteoporosis exhibited by patients with myeloma (36). The radiomics parameter Vertl_RLNonUni derived from the GLRLM reflects the heterogeneity of the images in different directions, and the Vertl_RLNonUni had been demonstrated in previous studies as a reliable indicator that could be used to predict the grading and staging of clear cell renal cell carcinoma and to perform risk stratification (37). In our study, it was hypothesized that this may be due to the different pathological mechanisms of MM and BM, as well as the fact that BM was an infiltrative lesion while MM was a diffuse lesion (38).

Our findings were highly reproducible because we applied rigorous subject screening and reliability testing of lesion segmentation during the study. The LASSO regression algorithm has been applied and validated many times in the past to have good utility in screening the feature parameters. Our radiomics model has been validated by methods such as the Calibration curve and DCA curve, and has good fitness and is very close to the ideal curve. It was worth noting that we performed the outline of ROI in 2D mode rather than 3D mode, and the radiomic features generated by different mode outlines may be different. Still, past studies had demonstrated that the models constructed in 2D or 3D mode achieved similar classification diagnostic performance (39). In this study, we compared the bone metastases of different malignant with MM, it is unclear whether the characteristic parameters of bone metastases caused by different malignancies are the same, Xiong et al. have tried to distinguish the BM characteristics of lung cancer and other cancers, and their results achieved only moderate diagnostic results in distinguishing them (20). In addition, patients with BM and MM may have altered images after chemotherapy or radiotherapy, such as focal radiotracer uptake and SUV measurements. Post-treatment lesions exhibiting flare phenomena and osteogenic-type responses may also introduce changes to the features extraction. Therefore, we strictly screened the enrolled patients to eliminate the effect of treatment on the images and ensure the rigor of this study. Finally, the use of pre-treatment images to construct radiomic models had the potential to help clinicians physicians to determine the sensitivity of patients to radiotherapy or chemotherapy and, thus, better stratify patients to determine more appropriate individualized treatment plans.

There were still exist some limitations in our study. First, as a single-center study, this study may be biased in terms of patient selection, and thus, the results may hardly represent generalizable findings. In addition, there was a lack of external validation data from the multicenter association. Therefore, all aspects still need to be adjusted and optimized before applying to the clinic. Second, retrospective studies may have selection bias related to study data collection. Third, not all patients enrolled had pathological findings, and we combined pathological findings and follow-up results to determine the classification of lesions under strict adherence to inclusion and exclusion criteria.

## 5. Conclusion

The radiomics model constructed based on 18F-FDG PET/CT images achieved satisfactory diagnostic performance for the classification of MM and bone metastases. In addition, the radiomics model showed a significant improvement in diagnostic performance compared to human experts and PET conventional parameter SUVmax. This non-invasive method could be used as a complement to traditional diagnostic methods. Furthermore, it had the potential to help clinicians physicians to develop individualized treatment plans, avoid adverse risks, and improve treatment outcomes.

## Data Availability Statement

The original contributions presented in the study are included in the article/Supplementary Material, further inquiries can be directed to the corresponding authors.

## Ethics Statement

Ethical review and approval was not required for the study on human participants in accordance with the local legislation and institutional requirements. Written informed consent from the patients/participants their next of kin was not required to participate in this study in accordance with the national legislation and the institutional requirements.

## Author Contributions

ZJ and JY contributed to the conception and design of the study. FZ and YIW carried out data statistics and analysis. ZJ wrote the manuscript. JY revised the manuscript. All authors read and approved the final manuscript.

## Funding

This study was supported by the Nature Science Foundation of Liaoning Province of China (No. 2019-ZD-0620).

## Conflict of Interest

The authors declare that the research was conducted in the absence of any commercial or financial relationships that could be construed as a potential conflict of interest.

## Publisher's Note

All claims expressed in this article are solely those of the authors and do not necessarily represent those of their affiliated organizations, or those of the publisher, the editors and the reviewers. Any product that may be evaluated in this article, or claim that may be made by its manufacturer, is not guaranteed or endorsed by the publisher.
