# Supplementary material for: Application of 18F-FDG PET-CT Images Based Radiomics in Identifying Vertebral Multiple Myeloma and Bone Metastases
Source: Front Med (Lausanne). 2022 Apr 18;9:874847. doi: 10.3389/fmed.2022.874847 (PMC9058063; doi:10.3389/fmed.2022.874847)
Supplement: Supplementary file 1 [file Data_Sheet_1.DOCX]

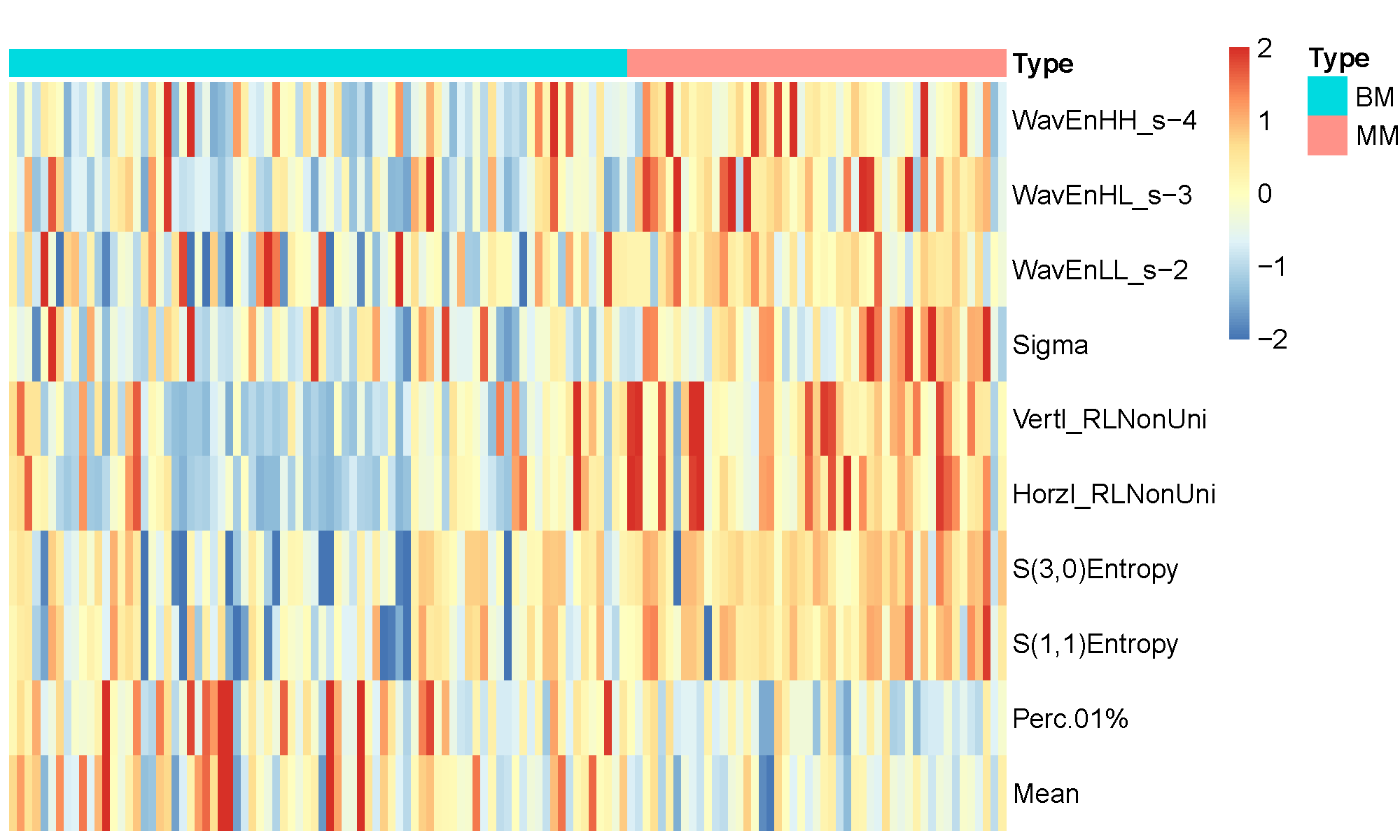


Figure S1: The heatmap of selected features for CT model of training group. The different shades of color represent the values of the features.


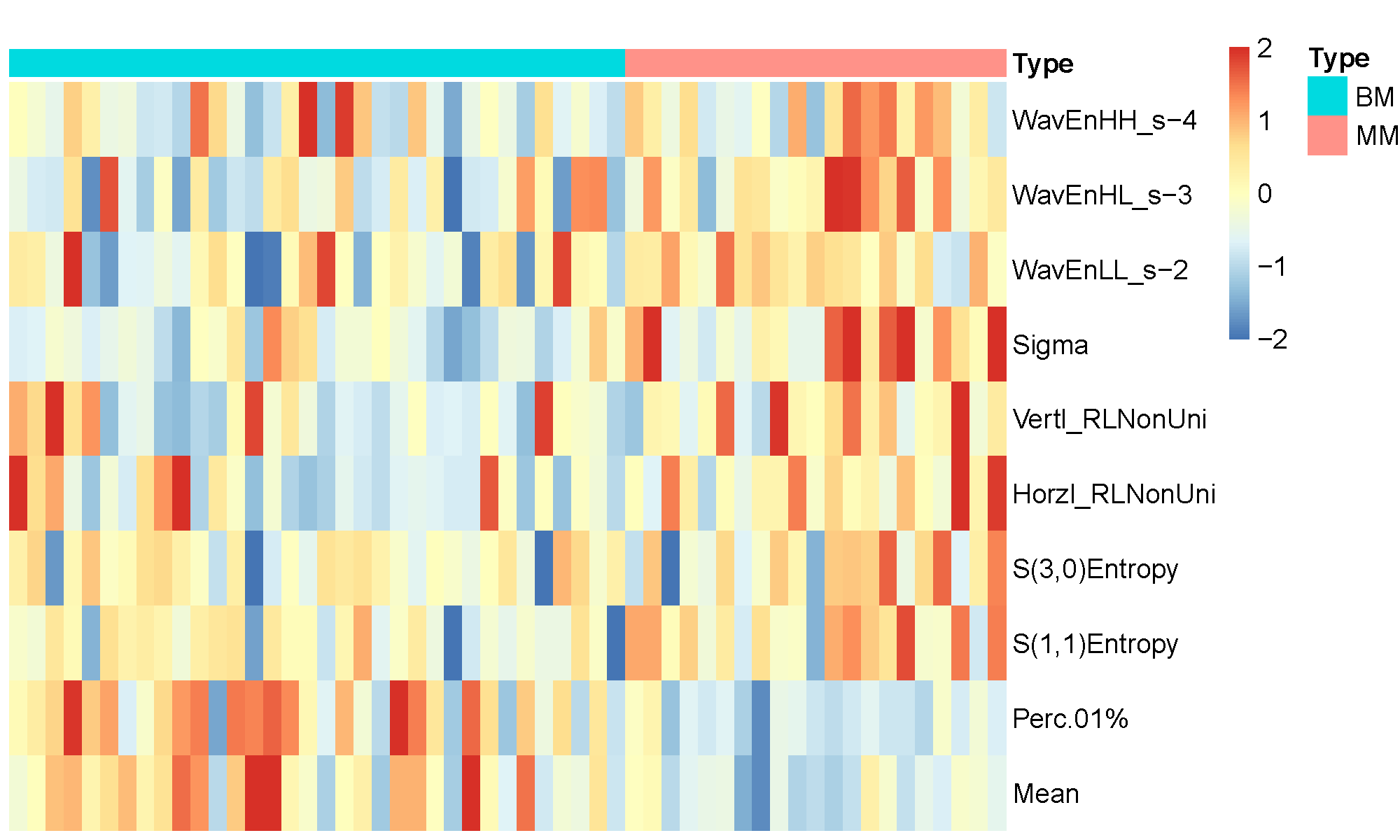


Figure S2: The heatmap of selected features for CT model of validation group.


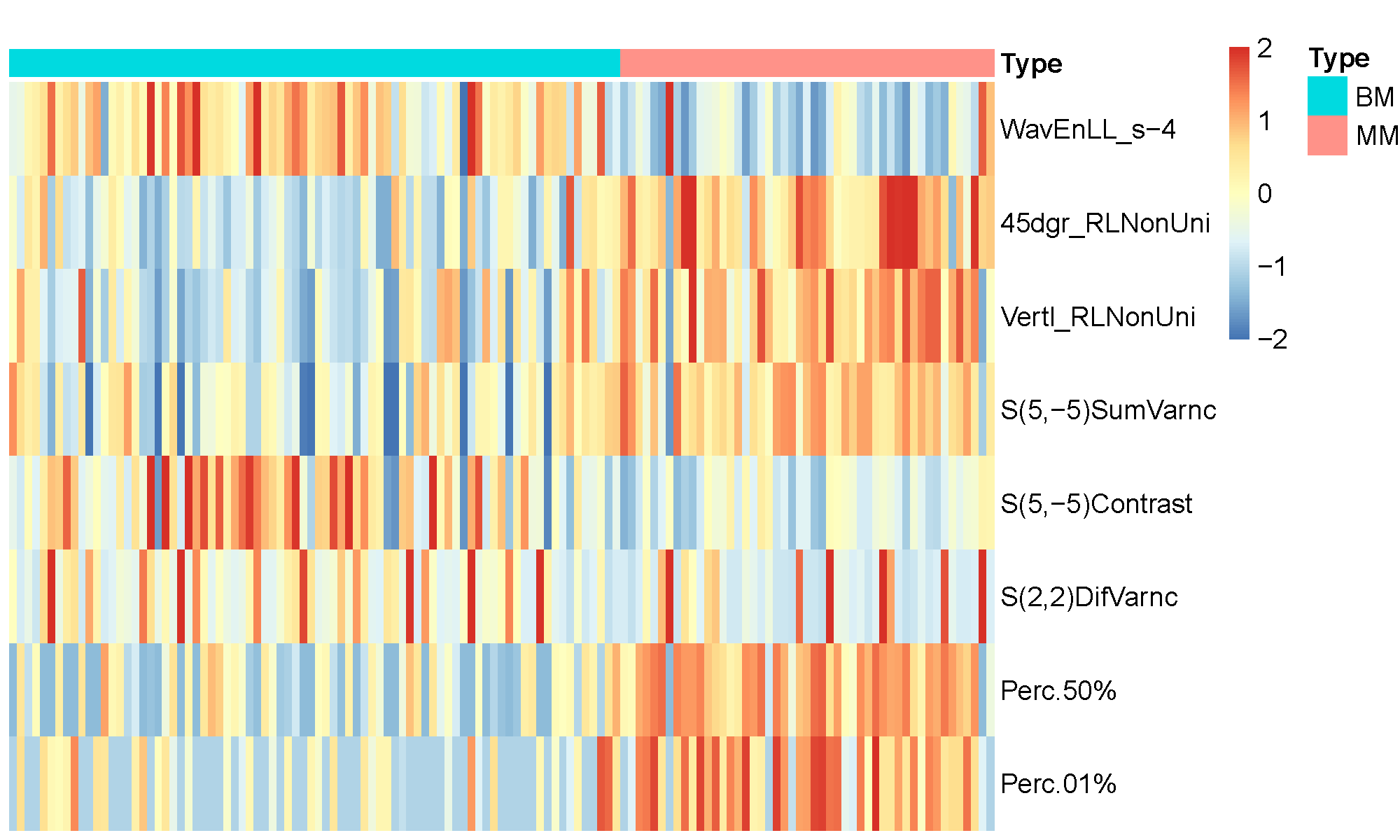


Figure S3: The heatmap of selected features for PET model of training group.


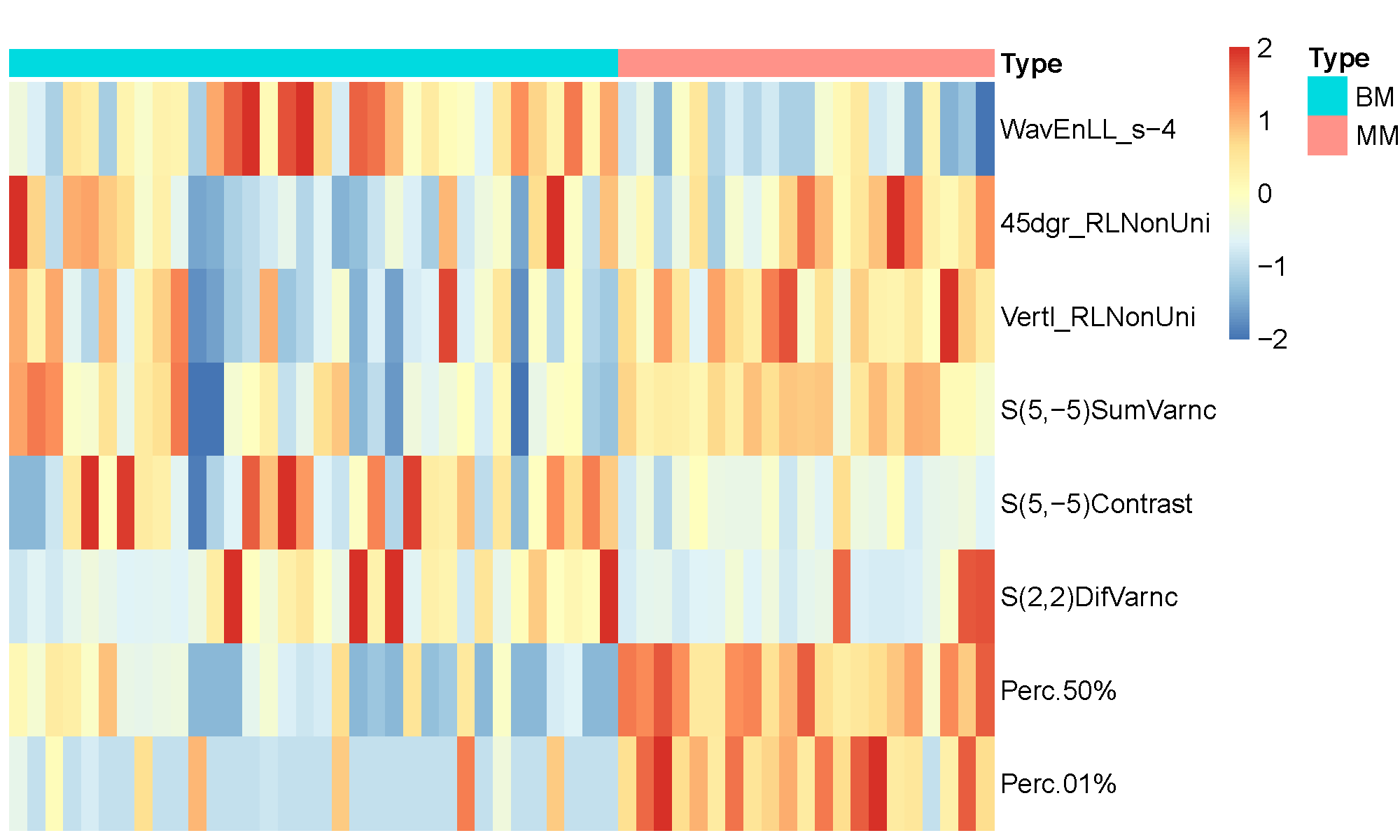


Figure S4: The heatmap of selected features for PET model of validation group.

Table A: Radiomics features contained in CT and PET models and their coefficients.

|  | **Feature** | **Coefficient** |
| --- | --- | --- |
| **CT**  **PET** | Intercept  WavEnLL_s-2  WavEnHL_s-3  WavEnHH_s-4  Sigma  Vertl_RLNonUni  Horzl_RLNonUni  S(1,1)Entropy  S(3,0)Entropy  Perc.01%  Mean  Intercept  WavEnLL_s-4  45dgr_RLNonUni  Vertl_RLNonUni  S(5,-5)SumVarnc  S(5,-5)Contrast  S(2,2)DifVarnc  Perc.50%  Perc.01% | 0.776299067  -0.330823668  -0.47072077  -0.099836985  -0.274955288  -0.683235453  -0.195541563  -0.364542816  -0.001775122  0.300112295  0.243807795  0.79927277  0.01024474  -0.75691117  -0.04571822  -0.35075169  0.51482754  -0.70561298  -1.00057195  -0.21800208 |
